# Short Sleep Duration: Children’s Mental, Behavioral, and Developmental Disorders and Demographic, Neighborhood, and Family Context in a Nationally Representative Sample, 2016–2019

**DOI:** 10.5888/pcd20.220408

**Published:** 2023-07-13

**Authors:** Angelika H. Claussen, Lina V. Dimitrov, Sivapriya Bhupalam, Anne G. Wheaton, Melissa L. Danielson

**Affiliations:** 1 Centers for Disease Control and Prevention, National Center on Birth Defects and Disabilities, Division of Human Development and Disability, Atlanta, Georgia; 2 Oak Ridge Institute for Science and Education, Oak Ridge, Tennessee; 3 Centers for Disease Control and Prevention, National Center for Chronic Disease Prevention and Health Promotion, Division of Population Health, Atlanta, Georgia

## Abstract

**Introduction:**

Many children and adolescents experience insufficient sleep, which poses risks for their short- and long-term health and development. This study examined the concurrent associations of contextual factors, including child, demographic, neighborhood, and family factors, with short sleep duration.

**Methods:**

We combined data on children aged 3 to 17 years from the 2016–2019 National Survey of Children's Health (N = 112,925) to examine the association of parent-reported child short sleep duration (ages 3–5 y, <10 h; 6–12 y, <9 h; 13–17 y, <8 h) with mental, behavioral, and developmental disorders (MBDDs); selected physical health conditions; and demographic, neighborhood, and family factors.

**Results:**

Overall, 34.7% of children experienced short sleep duration. The prevalence was highest among children aged 6 to 12 years (37.5%); children from racial and ethnic minority groups, especially non-Hispanic Black children (50.0%); children from low-income households (44.9%); children with an MBDD (39.6%); children experiencing negative neighborhood factors (poor conditions and lack of safety, support, and amenities, 36.5%); and family factors such as inconsistent bedtime (57.3%), poor parental mental (47.5%) and physical health (46.0%), and adverse childhood experiences (44.1%). The associations between sleep and demographic, neighborhood, and family factors, and MBDD remained significant after controlling for all other factors.

**Conclusion:**

This study identified several individual, family, and community factors that may contribute to children’s short sleep duration and can be targeted to improve healthy development, particularly among children with an MBDD, from households with low socioeconomic status, or from racial and ethnic minority groups who are at increased risk for short sleep duration.

**Summary**

**What is already known on this topic?**

Many children and adolescents experience insufficient sleep. Short sleep duration among children is associated with mental, behavioral, and developmental disorders, is influenced by demographic, neighborhood, and family contexts, and poses risks for long-term health and development.

**What is added by this report?**

We examined these factors concurrently and showed the significant and independent association of childhood disorders and demographic, neighborhood, and family factors with short sleep duration.

**What are the implications for public health practice?**

Short sleep duration is more prevalent among children with mental, behavioral, and developmental disorders, in racial and ethnic minority groups, and from low-socioeconomic households. We identified a range of neighborhood and family factors that can be targeted to improve children’s sleep and promote healthy development.

## Introduction

Sleep is an essential function that predicts long-term health and well-being (1). Insufficient sleep has been associated with poor physical health (1–3), poor mental health, and problems with attention, behavior, learning, and memory (1,4–6). Insufficient sleep includes poor sleep quality and short sleep duration. This study focused on short sleep duration, which is common among children: during 2016–2018, 35% of US children aged 0 to 17 years had shorter sleep duration than recommended for their age (1) based on parent report (7). However, research on sleep among children is less extensive than among adults (4,8).

Child, family, and environmental factors can all influence sleep (5,9,10). Sleep is negatively influenced by internal and external signals of danger and disruption (11). Psychological stressors within the family, such as parental health and well-being (12–15), and psychological stressors in the social context, such as neighborhood disadvantage and lack of perceived safety, affect sleep (9,10,16,17). Sleep is also affected by child characteristics, such as special health care needs and mental disorders (7,18), and family factors, such as regular bedtimes (7,19,20).

Children experiencing poverty are at increased risk for insufficient sleep (7,21–23). Poverty is related to risks that impair sleep, such as health risks, danger and stress, crowding, lack of suitable sleep spaces, or food insecurity (9,10,15). Children from racial and ethnic minority groups experience increased risk for insufficient sleep (7,9,21,22,24), potentially through mental stress from experiencing racism and discrimination (9,23), and through structural racism and the associated higher prevalence of socioeconomic risks related to poor sleep (21,22).

Previous studies examined the association between sleep and factors such as mental health and environmental factors, and between social context and children’s mental health, but not comprehensively in a single sample of young and older children. Given that demographic, social, and environmental risk factors co-occur, understanding the independent associations between different types of risks and poor sleep requires examination of these different risk factors in a comprehensive data set. Our study expands on previous research (7) that documented short sleep duration in a nationally representative sample of children and adolescents. We examined concurrent and independent associations between demographic, social, and environmental factors, mental disorders, and sleep duration in this comprehensive data set based on a nationally representative sample of children and adolescents to identify potential targets for public health intervention.

## Methods

We used 4 years (2016–2019) of data from the National Survey of Children’s Health, a nationally representative survey administered by the US Census Bureau and funded and directed by the Maternal and Child Health Bureau of the Health Resources and Services Administration (25). The survey uses a combination of an online and a pencil-and-paper format. Respondents are parents (including other caregivers or guardians who are familiar with the child’s health and health care, hereinafter referred to as “parents”) reporting on a single child aged 0 to 17 years per household.

We used guidance provided by the US Census Bureau (26) to combine data on 131,774 children aged 0 to 17 years from the 4 survey years. We used data from before the COVID-19 pandemic, because pandemic-related factors likely affected sleep duration and other indicators of interest. The overall weighted response rates were 41% (2016), 37% (2017), 43% (2018), and 42% (2019). We excluded children aged 0 to 2 years (n = 17,298) and children without valid data on sleep duration (n = 1,551). The final analytic sample size was 112,925 (Figure).

**Figure:**
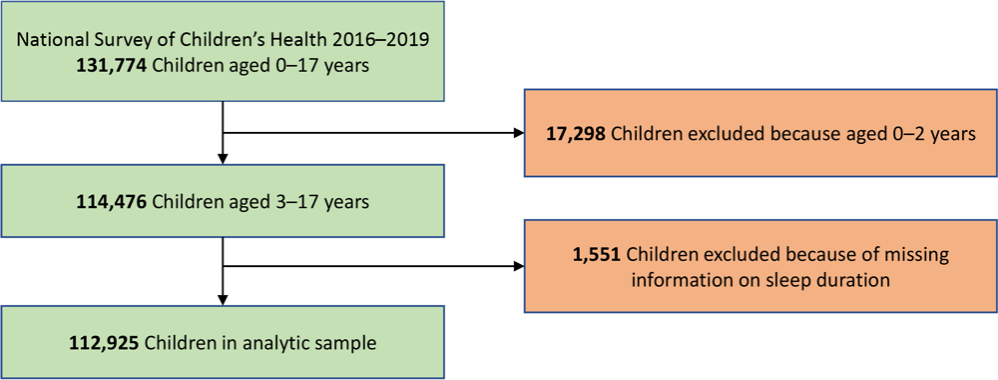
Sample size and exclusion and inclusion criteria for the analytic sample, the National Survey of Children’s Health, 2016–2019.

### Sleep indicator

Parents of children aged 3 to 5 years were asked, “During the past week, how many hours of sleep did this child get on an average day (count both nighttime sleep and naps)?” For ages 6 to 17 years, the question was, “During the past week, how many hours of sleep did this child get on an average weeknight?” in 2016 and 2017 and “on most weeknights?” in 2018 and 2019. On the basis of recommendations for sleep duration from the American Academy of Sleep Medicine, the survey defined short sleep duration as less than 10 hours for ages 3 to 5 years (preschool age), less than 9 hours for ages 6 to 12 years (school age), and less than 8 hours for ages 13 to 17 years (adolescents) (25).

### Demographic, neighborhood, and family factors

Parents reported on demographic characteristics such as the child’s sex, age, race and ethnicity, parental education (highest level of education attained by primary caregivers), and household size and family income. Data on household size and income were used by the US Census Bureau to calculate family income as a percentage of the federal poverty level. Parents also reported on neighborhood factors such as safety, support, condition, and amenities. Negative factors (unsafe neighborhood, neighborhood lacks support, neighborhood in bad condition, and neighborhood lacks amenities) were combined into a single composite variable labelled “negative neighborhood factors.” Family factors included whether the child had a consistent bedtime, child’s exposure to adverse childhood experiences (ACEs), and parental mental and physical health status.

### Mental, behavioral, and developmental disorders

Parents were asked whether a health care provider had ever told them that their child had any of a series of disorders, including attention-deficit and hyperactivity disorder (ADHD), behavior problems, anxiety, depression, Tourette syndrome, learning disability, developmental delay, intellectual disability, speech or language disorder, and autism spectrum disorder and whether the child currently had the disorder; behavior problems, learning disability, developmental delay, intellectual disability, and speech or language disorder could also be identified by an educator. Current ADHD, behavior problems, anxiety, depression, and Tourette syndrome were grouped into the category MEB (mental, emotional, and behavioral disorders). Current learning disability, developmental delay, intellectual disability, speech or language disorder, and autism spectrum disorder were combined into the category DLLD (developmental, language, or learning disorders). MEB was combined with DLLD into the category MBDD (mental, behavioral, and developmental disorders). Parents also reported children’s physical health conditions.

### Analyses

We examined the relationship between short sleep duration and selected demographic characteristics (sex, age group, race and ethnicity, parental education, and family income), and presence of MBDD and physical health conditions. We also examined negative neighborhood and family factors, including inconsistent bedtime, parental mental and physical health, and number of ACEs for the child. For the publicly available data set, imputed values for missing data on family income (17.1% missing) were calculated via multiple imputation; imputed values for missing data on parental education (2.1% missing) and children’s sex (0.1% missing), race (0.4% missing), and Hispanic origin (0.6% missing) were calculated via hot-deck imputation. Respondents with missing values for any other factors were excluded from item-level analyses (missingness was 3.5% or less per item). Given the minor wording change in the sleep item for children aged 6 to 17 years after 2017, we conducted sensitivity analyses to ensure that patterns of associations were similar across survey years. These analyses did not show meaningful differences across study years; therefore, we analyzed the sample as a whole.

We conducted all analyses in SAS-callable SUDAAN version 11.0.1 (RTI International) to account for the complex survey design and weighting of the data. We used individual logistic regression models with predicted marginals to calculate weighted prevalence ratios (PRs) and 95% confidence intervals (CIs) to compare the prevalence of short sleep duration among children and adolescents aged 3 to 17 years across demographic subgroups, presence or absence of neighborhood or family risk factors, and presence of any MBDD. We ran logistic regression models to produce adjusted prevalence ratios (aPRs) for the association between each MEB or presence of any DLLD or physical health condition and short sleep duration adjusted for sociodemographic, neighborhood, and family risk factors; an adjusted model of the association between any MBDD and short sleep duration also produced aPRs for all variables included in the model. To examine whether each of the demographic, family, and neighborhood characteristics were independently associated with short sleep duration when considered together, we calculated the PRs of short sleep duration for each characteristic in the adjusted model predicting short sleep duration based on MBDD status. All prevalence estimates and 95% CIs met the National Center for Health Statistics Data Presentation Standards (27).

## Results

### Demographic factors

Among children aged 3 to 17 years, 34.7% (95% CI, 34.1%–35.4%) were reported to experience short sleep duration. Sleep duration was associated with child’s age; parents reported short sleep most frequently for children aged 6 to 12 years (37.5%; 95% CI, 36.5%–38.5%) and least for adolescents aged 13 to 17 years (30.7%; 95% CI, 29.6%–31.7%) (Table 1).

**Table 1:** Weighted Prevalence of Short Sleep Duration Among Children Aged 3–17 Years, by Demographic, Neighborhood, and Family Characteristics, National Survey of Children’s Health, United States, 2016–2019

Characteristic	No. of children	Short sleep duration,^a^ weighted % (95% CI)	Prevalence ratio (95% CI)
**Individual demographic factors**			
**Sex** b			
Male	58,341	35.1 (34.1–36.0)	1.02 (0.98–1.06)
Female	54,584	34.4 (33.4–35.3)	1 [Reference]
**Age, y**			
3–5	19,966	35.0 (33.5–36.6)	1.14 (1.08–1.21)
6–12	46,897	37.5 (36.5–38.5)	1.22 (1.17–1.28)
13–17	46,062	30.7 (29.6–31.7)	1 [Reference]
**Race and ethnicity** b			
Hispanic	12,885	38.6 (36.7–40.5)	1.33 (1.26–1.40)
Non-Hispanic American Indian/Alaska Native	665	37.5 (31.2–44.2)	1.29 (1.09–1.53)
Non-Hispanic Asian	5,767	32.7 (30.2–35.1)	1.12 (1.04–1.21)
Non-Hispanic Black	7,048	50.0 (47.9–52.0)	1.72 (1.64–1.80)
Non-Hispanic Native Hawaiian/Pacific Islander	277	36.3 (25.5–48.2)	1.25 (0.93–1.69)
Non-Hispanic White	78,733	29.0 (28.4–29.6)	1 [Reference]
Non-Hispanic other (2016–2018 only)	647	36.1 (29.3–43.3)	1.24 (1.03–1.50)
Non-Hispanic ≥2 races	6,903	33.5 (31.2–35.8)	1.15 (1.07–1.24)
**Parental education^b^**			
Less than high school	2,680	45.6 (42.1–49.1)	1.66 (1.53–1.80)
High school	14,570	42.5 (40.8–44.3)	1.55 (1.48–1.63)
Some college or associate degree	26,325	38.6 (37.3–39.9)	1.41 (1.35–1.47)
College degree or higher	68,484	27.4 (26.7–28.2)	1 [Reference]
**Family income, % of FPL** c			
<100%	12,371	44.9 (43.0–46.8)	1.73 (1.63–1.82)
100% to ≤199%	18,205	39.3 (37.5–41.0)	1.51 (1.43–1.59)
200% to ≤399%	34,700	33.4 (32.2–34.7)	1.29 (1.22–1.35)
≥400%	47,649	26.0 (25.2–26.9)	1 [Reference]
**Negative neighborhood factors**			
**Overall**			
≥1 Negative neighborhood factors^d^	83,770	36.5 (35.7–37.3)	1.28 (1.22–1.34)
No negative neighborhood factors	27,901	28.5 (27.3–29.7)	1 [Reference]
**Safety**			
Unsafe neighborhood^e^	3,589	46.2 (42.7–49.7)	1.36 (1.26–1.47)
Safe neighborhood	107,376	34.0 (33.3–34.7)	1 [Reference]
**Support**			
Neighborhood lacks support^f^	23,813	40.6 (39.1–42.0)	1.25 (1.20–1.30)
Neighborhood provides support	86,446	32.4 (31.7–33.2)	1 [Reference]
**Condition**			
Neighborhood in bad condition^g^	23,196	40.3 (38.8–41.9)	1.24 (1.19–1.29)
Neighborhood in good condition	87,560	32.6 (31.9–33.3)	1 [Reference]
**Amenities**			
Neighborhood lacks amenities^h^	70,140	35.8 (35.0–36.7)	1.10 (1.06–1.15)
Neighborhood has amenities	40,522	32.6 (31.5–33.7)	1 [Reference]
**Family factors**			
**Consistency of bedtime**			
Inconsistent^i^	12,448	57.3 (55.4–59.2)	1.82 (1.75–1.90)
Consistent	100,103	31.4 (30.7–32.1)	1 [Reference]
**Mental health of parent**			
≥1 Parent with fair/poor mental health	7,988	47.5 (45.0–50.1)	1.43 (1.35–1.51)
No parent with fair/poor mental health^j^	101,120	33.3 (32.6–34.0)	1 [Reference]
**Physical health of parent**			
≥1 Parent with fair/poor physical health	10,269	46.0 (43.7–48.3)	1.40 (1.32–1.47)
No parent with fair/poor physical health^k^	98,946	33.0 (32.3–33.7)	1 [Reference]
**No. of ACEs** l			
0	64,843	28.9 (28.1–29.8)	1 [Reference]
1	24,776	39.2 (37.7–40.6)	1.35 (1.29–1.42)
2	10,517	42.1 (40.0–44.2)	1.45 (1.37–1.54)
3	5,305	44.4 (41.3–47.4)	1.53 (1.42–1.65)
≥4	6,364	47.2 (44.4–49.9)	1.63 (1.53–1.74)

Abbreviations: ACEs, adverse childhood experiences; FPL, federal poverty level.

a For children aged 3–5 years: “During the past week, how many hours of sleep did this child get during an average day (count both nighttime sleep and naps)?” For children aged 6–17 years for 2018–2019: “During the past week, how many hours of sleep did this child get on most weeknights.” The question in 2016–2017 asked about “an average weeknight.” Short sleep duration was defined as <10 h for children aged 3–5 years, <9 h for children aged 6–12 years, and <8 h for children aged 13–17 years.

b If missing, this variable was imputed by using hot-deck imputation methods.

c If missing, family income was imputed by using sequential regression as an input to FPL; FPL was multiply imputed and contained 6 implicates.

d Category comprised 4 subcategories: “unsafe neighborhood,” “neighborhood lacks support,” “neighborhood in bad condition,” and “neighborhood lacks amenities.” Endorsement of any of these subcategories was counted as a respondent having ≥1 negative neighborhood factor.

e A response of definitely disagree/somewhat disagree (vs somewhat agree/definitely agree) when asked whether the child is safe in their neighborhood.

f A response of definitely disagree/somewhat disagree (vs somewhat agree/definitely agree) when asked whether people in the neighborhood help each other out, watch out for each other’s children, and know where to go for help in their community when they encounter difficulties.

g A response of yes to any of these 3 items: 1) litter or garbage on the street or sidewalk, 2) poorly kept or rundown housing, or 3) vandalism such as broken windows or graffiti in their neighborhood.

h A response of no to any of these 4 items: 1) sidewalks or walking paths, 2) a park or playground, 3) a recreation center, community center, or boys’ and girls’ club, and 4) a library or bookmobile in the neighborhood.

i A response of child never/rarely/sometimes (vs usually/always) goes to bed at the same time on weeknights.

j Parent (both parents if 2 primary caregivers) reported fair/poor (vs good/very good/ excellent) mental health.

k Parent (both parents if 2 primary caregivers) reported fair/poor (vs good/very good/excellent) physical health.

l Child’s ACEs included 9 items: 1) lived in a household where it was very hard to cover the basics, like food or housing, on the family’s income; 2) parent or guardian divorced; 3) parent or guardian died; 4) parent or guardian served time in jail; 5) child saw or heard parents or adults slap, hit, kick, punch one another in the home; 6) was victim of violence or witnessed violence in neighborhood; 7) lived with anyone who was mentally ill, suicidal, or severely depressed, or 8) anyone who had a problem with alcohol or drugs; and 9) treated or judged unfairly because of his or her race or ethnic group. Data on ACEs related to child maltreatment were not available because the survey was based on parent report.

We found racial and ethnic disparities in sleep duration. Compared with non-Hispanic White children, children from each of the other racial and ethnic groups had significantly higher prevalence of short sleep duration, except for the non-Hispanic Native Hawaiian and Pacific Islander group. For example, the prevalence of short sleep duration among non-Hispanic White children was 29.0%, 38.6% among Hispanic children, and 50.0% among non-Hispanic Black children.

Short sleep duration was associated with lower parental education level; children of parents with less than a high school education had the highest prevalence of short sleep duration (45.6%), and children of parents with college degrees or higher had the lowest (27.4%). Similar patterns emerged for family income. The highest percentage of short sleep duration (44.9%) was among children in families with the lowest income level (<100% of the federal poverty level). Estimates of short sleep duration decreased as income levels increased and differed among all 4 levels. Sex was not associated with short sleep duration even when stratified by age.

### Neighborhood factors

All 4 negative neighborhood factors (unsafe neighborhood, neighborhood lacks support, neighborhood in bad condition, and neighborhood lacks amenities) were significantly associated with a higher prevalence of short sleep duration; 36.5% of children living in neighborhoods with 1 or more negative neighborhood factors experienced short sleep duration, compared with 28.5% living in neighborhoods without negative factors.

### Family factors

Short sleep duration was experienced by 57.3% of children with an inconsistent bedtime. Having a parent with fair or poor mental health or physical health and having a higher number of ACEs were also associated with significantly higher prevalence of short sleep duration.

### Disorders

The overall association of any MBDD with short sleep duration was significant (any MBDD, 39.6% vs no MBDD, 33.2%). The association also held true when comparing children with no MBDDs against those with MEBs together (40.9%), or against those with ADHD (40.3%), behavior problems (44.6%), anxiety (40.9%), and depression (48.0%) separately (Table 2). The association with Tourette syndrome was not significant. Having any DLLD was also associated with short sleep duration (38.6%) as was any physical health condition (36.1% vs 34.0% with no physical health condition).

**Table 2:** Weighted Prevalence of Short Sleep Duration Among Children Aged 3–17 Years, by Current Diagnoses, National Survey of Children’s Health, United States, 2016–2019

Diagnosis	No. of children with disorder or condition^a^	Short sleep duration,^b^ weighted % (95% CI)	Unadjusted PR (95% CI)	Adjusted PR^c^ (95% CI)
**Mental, behavioral, or developmental disorder (MBDD)** d				
No MBDD	83,772	33.2 (32.5–34.0)	1 [Reference]	1 [Reference]
Any MBDD^e^	26,288	39.6 (38.2–40.9)	1.19 (1.14–1.24)	1.06 (1.02–1.11)
Any mental, emotional, or behavioral disorder^f^	20,974	40.9 (39.4–42.4)	1.23 (1.18–1.28)	1.11 (1.06–1.16)
ADHD	11,370	40.3 (38.4–42.2)	1.21 (1.15–1.28)	1.08 (1.02–1.15)
Behavior problems	8,220	44.6 (42.3–47.0)	1.34 (1.27–1.42)	1.10 (1.03–1.17)
Anxiety	11,053	40.9 (38.9–42.9)	1.23 (1.17–1.30)	1.14 (1.08–1.21)
Depression	4,845	48.0 (44.8–51.1)	1.44 (1.35–1.55)	1.24 (1.14–1.34)
Tourette syndrome	275	39.4 (27.1–52.9)	1.19 (0.87–1.63)	1.39 (1.13–1.71)
Any developmental, learning, or language disorders^g^	12,812	38.6 (36.7–40.6)	1.16 (1.10–1.23)	1.00 (0.94–1.06)
**Physical health conditions^h^**				
No physical health condition	72,361	34.0 (33.2–34.8)	1 [Reference]	1 [Reference]
Any physical health condition	36,956	36.1 (35.0–37.2)	1.06 (1.02–1.10)	1.00 (0.97–1.04)

Abbreviations: ADHD, attention-deficit/hyperactivity disorder; PR, prevalence ratio.

a Categories of mental, behavioral, or developmental disorders are not mutually exclusive; children with multiple conditions are included in each applicable category.

b For children aged 3–5 years: “During the past week, how many hours of sleep did this child get during an average day (count both nighttime sleep and naps)?” For children aged 6–17 years for 2018–2019: “During the past week, how many hours of sleep did this child get on most weeknights.” The question in 2016–2017 asked about “an average weeknight.” Short sleep duration was defined as <10 h for children aged 3–5 years, <9 h for children aged 6–12 years, and <8 h for children aged 13–17 years.

c Adjusted PR calculated by controlling for sex, age, race and ethnicity, parental education, poverty, neighborhood factor composite (ie, ≥1 negative neighborhood factors vs no negative neighborhood factors), inconsistent bedtime, ≥1 parent with fair/poor mental health, ≥1 parent with fair/poor physical health, and ACEs.

d For each analysis in each group of MBDDs, children with a specific disorder were compared with children without any MBDDs. 83,772 children had no MBDDs; 2,865 children did not have information on all MBDDs and were not included in this analysis.

e A response of yes to current mental, behavioral, or developmental disorder (includes attention-deficit/hyperactivity disorder), behavior problems, anxiety, depression, learning disability, developmental delay, intellectual disability, speech and language disorder, autism spectrum disorder, and Tourette syndrome.

f A response of yes to current mental, emotional, or behavioral disorder (includes attention-deficit/hyperactivity disorder, behavior problems, anxiety, depression, Tourette syndrome).

g A response of yes to current developmental, language, or learning disorder (includes learning disability, developmental delay, intellectual disability, speech and language disorder, and autism spectrum disorder).

h A response of yes to current allergies, arthritis, asthma, blood disorder, brain injury, cerebral palsy, cystic fibrosis, diabetes, Down syndrome, epilepsy, heart condition, headaches, deafness, or blindness.

After adjustment for demographic, family, and neighborhood characteristics, having an MBDD remained significantly associated with short sleep duration. This pattern was similar for MEB as a group and for ADHD, behavior problems, anxiety, and depression separately. The association with Tourette syndrome was significant in the adjusted model. The association of a DLLD diagnosis and having any physical health condition was no longer significant in the adjusted models (Table 2).

The magnitude of the association between short sleep duration and age was larger in the adjusted models than in the bivariate models, especially among children aged 6 to 12 years (Table 3). For race and ethnicity, only Hispanic (aPR = 1.12), non-Hispanic Black (aPR = 1.44), and non-Hispanic Asian (aPR = 1.15) children still had a significantly higher prevalence of short sleep duration than non-Hispanic White children after adjustment. Parental education, family income level, neighborhood factors, parent mental and physical health, and child’s ACEs remained significantly associated with short sleep duration, but the magnitude of the association was smaller than in bivariate models. The aPR for inconsistent bedtime remained the highest among the factors in the adjusted models, only slightly reduced from 1.82 to 1.70.

**Table 3:** Independent Contribution of Children’s Mental, Behavioral, or Developmental Disorders, Demographic, Neighborhood, and Family Factors to Short Sleep Duration, National Survey of Children’s Health, United States, 2016–2019

Variable	Short sleep duration,^a^ adjusted prevalence ratio (95% CI)^b^
**Mental, behavioral, or developmental disorders**	
None	1 [Reference]
Any^c^	1.06 (1.02–1.11)
**Demographic characteristics**	
**Sex** d	
Male	1.01 (0.98–1.05)
Female	1 [Reference]
**Age, y**	
3–5	1.29 (1.22–1.36)
6–12	1.33 (1.27–1.38)
13–17	1 [Reference]
**Race and ethnicity** d	
Hispanic	1.12 (1.06–1.19)
Non-Hispanic American Indian/Alaska Native	1.00 (0.82–1.21)
Non-Hispanic Asian	1.15 (1.06–1.24)
Non-Hispanic Black	1.44 (1.36–1.51)
Non-Hispanic Native Hawaiian/Pacific Islander	1.03 (0.76–1.39)
Non-Hispanic White	1 [Reference]
Non-Hispanic other (2016–2018 only)	1.05 (0.85–1.29)
Non-Hispanic ≥2 races	1.05 (0.98–1.12)
**Parental education** d	
Less than high school	1.34 (1.22–1.48)
High school	1.25 (1.18–1.32)
Some college or associate degree	1.18 (1.13–1.24)
College degree or higher	1 [Reference]
**Family income level, % of FPL** e	
<100%	1.12 (1.04–1.20)
100% to ≤199%	1.08 (1.01–1.15)
200% to ≤399%	1.07 (1.02–1.12)
≥400%	1 [Reference]
**Negative neighborhood factors** f	
≥1	1.10 (1.05–1.15)
None	1 [Reference]
**Family factors**	
**Consistency of bedtime**	
Inconsistent^g^	1.70 (1.63–1.78)
Consistent	1 [Reference]
**Mental health of parent**	
≥1 Parent with fair/poor mental health	1.09 (1.01–1.17)
No parent with fair/poor mental health^h^	1 [Reference]
**Physical health of parent**	
≥1 Parent with fair/poor physical health	1.09 (1.02–1.16)
No parent with fair/poor physical health^i^	1 [Reference]
**No. of ACEs^j^**	
0	1 [Reference]
1	1.19 (1.13–1.25)
2	1.22 (1.15–1.30)
3	1.25 (1.15–1.35)
≥4	1.28 (1.19–1.39)

Abbreviation: ACEs, adverse childhood experiences; FPL, federal poverty level.

a For children aged 3–5 years: “During the past week, how many hours of sleep did this child get during an average day (count both nighttime sleep and naps)?” For children aged 6–17 years for 2018–2019: “During the past week, how many hours of sleep did this child get on most weeknights.” The question in 2016–2017 asked about “an average weeknight.” Short sleep duration was defined as <10 h for children aged 3–5 years, <9 h for children aged 6–12 years, and <8 h for children aged 13–17 years.

b Adjusted prevalence ratio calculated by controlling for all other factors in the model: sex, age, race and ethnicity, parental education, poverty, neighborhood factor composite (ie, ≥1 negative neighborhood factors vs no negative neighborhood factors), inconsistent bedtime, ≥1 parent with fair/poor mental health, ≥1 parent with fair/poor physical health, ACEs, and mental, behavioral, or developmental disorders.

c A response of yes to current mental, behavioral, or developmental disorder (includes attention-deficit/hyperactivity disorder), behavior problems, anxiety, depression, learning disability, developmental delay, intellectual disability, speech and language disorder, autism spectrum disorder, and Tourette syndrome.

d If missing, this variable was imputed by using hot-deck imputation methods.

e If missing, family income was imputed by using sequential regression as an input to the FPL; FPL was multiply imputed and contained 6 implicates.

f Category comprised 4 subcategories: “unsafe neighborhood,” “neighborhood lacks support,” “neighborhood in bad condition,” and “neighborhood lacks amenities.” Endorsement of any of these subcategories was counted as a respondent having ≥1 negative neighborhood factor.

g A response of child never/rarely/sometimes (vs usually/always) goes to bed same time on weeknights.

h Parent (both parents if 2 primary caregivers) reported fair/poor (vs good/very good/ excellent) mental health.

i Parent (both parents if 2 primary caregivers) reported fair/poor (vs good/very good/ excellent) physical health.

j Child’s ACEs included 9 items: 1) lived in a household where it was very hard to cover the basics, like food or housing, on the family’s income; 2) parent or guardian divorced; 3) parent or guardian died; 4) parent or guardian served time in jail; 4) child saw or heard parents or adults slap, hit, kick, punch one another in the home; 6) was victim of violence or witnessed violence in neighborhood; 7) lived with anyone who was mentally ill, suicidal, or severely depressed, or 8) anyone who had a problem with alcohol or drugs; and 9) treated or judged unfairly because of his or her race or ethnic group. Data on ACEs related to child maltreatment were not available because the survey was based on parent report.

## Discussion

Our data show that parent-reported short sleep duration is common for children of all ages. Similar to data in previous reports (7), more than 1 in 3 children did not meet sleep recommendations during 2016–2019. The highest proportion of parent-reported short sleep duration was among children aged 6 to 12 years, with almost 2 in 5 children not meeting recommendations. Among adolescents, almost 1 in 3 had parent-reported short sleep duration.

In previous studies, a majority of adolescents self-reported shorter sleep duration than recommended for their age (28,29). Because parents may overestimate sleep, particularly among older children and adolescents, our data, based on parent report, may have conservatively estimated short sleep duration (5).

Having an inconsistent bedtime has been documented as affecting sleep duration (7,20), and was particularly notable among factors measured in our study. Our data showed that children with inconsistent bedtimes had about a 70% higher prevalence of insufficient sleep, even after controlling for child, family, and neighborhood factors.

As expected, sleep disparities were associated with social determinants of health (5,14,21,23,24). Short sleep duration was more prevalent among children in most racial and ethnic minority groups than among non-Hispanic White children (23,29). Non-Hispanic Black children had the highest prevalence and were approximately 50% more likely than non-Hispanic White children to have short sleep duration. We also found significant levels of short sleep duration among groups such as non-Hispanic Asian and American Indian or Alaskan Native children, or those with multiple races, whose sleep duration has previously not been well documented. Although associations decreased after adjusting for other demographic, family, and neighborhood factors and MBDD, children from several racial and ethnic minority groups still had significantly higher prevalence of short sleep duration, particularly non-Hispanic Black children. Given that racial and ethnic minority populations are affected by additional risks for health disparities that were not addressed in the available survey data, a more comprehensive examination of systemic racism and social determinants of health may be needed to understand what contributes to these sleep disparities (9,23,30). In our study, the child’s sex was not associated with short sleep duration, unlike in another study, which found shorter sleep among female adolescents (24).

As previously documented (9,14,22,24), children from households with lower socioeconomic status, as indicated by parental education and poverty levels, had higher percentages of short sleep duration. Neighborhood factors such as poor condition, and lack of amenities, safety, and support also were associated with short sleep duration, similar to previous studies (10,22,30). Short sleep duration among children was also associated with having parents with poor mental and physical health and with experiencing adversity in childhood (10,14,18,22,31).

Children with MBDD overall and MEB, including ADHD, behavior problems, anxiety, and depression specifically, had a significantly higher prevalence of short sleep duration than children without these disorders. The association with having an MBDD overall, and with each MEB disorder, was significant after controlling for demographic, neighborhood, and family factors. Having a developmental, language, or learning disorder or a physical health condition was also associated with short sleep duration; however, the associations were no longer significant after controlling for demographic, neighborhood, and family factors. Thus, this study provides further evidence that insufficient sleep is a concern among children with mental, emotional, and behavioral disorders (6,18). Associations between sleep and mental health can be bidirectional; mental health symptoms such as those experienced with ADHD, anxiety, or behavior problems can contribute to sleep problems and can also be exacerbated by insufficient sleep (1,4,6).

Given the increased probability of demographic, neighborhood, and family risk factors occurring together and being associated with children’s mental health (10,11,16), we were able to examine whether these factors contributed independently. Our data showed that MBDD was associated with short sleep duration even after adjusting for the influence of other contextual factors. We also examined each child, family, and neighborhood factors’ independent contribution to short sleep duration (controlling for MBDD diagnosis and all demographic, neighborhood, and family covariates) and found that, other than the child’s sex, each contributed to the probability that a child would experience short sleep duration.

Evidence that children’s MBDD and many of these demographic, family, and neighborhood variables are associated independently with short sleep duration points to potentially selecting these factors for prevention of insufficient sleep, particularly among communities affected by health disparities (16). Prior work suggests that sleep problems may also be a potential mechanism through which socioeconomic risk is translated into mental health problems (14,23). Further studies can explore whether comprehensive intervention approaches that improve family and community risks may improve both mental health and sleep, and thus, improve long-term healthy development.

### Strengths and limitations

This study has several strengths. The large, nationally representative data set allowed for consideration of more factors and more generalizable results than clinical samples would allow. We were able to investigate differences between racial and ethnic groups such as non-Hispanic Asian, non-Hispanic American Indian and Alaskan Native, and non-Hispanic Native Hawaiian and Pacific Islander, whose sleep duration and associated risks are often not evaluated separately in studies because of small cell sizes. The comprehensive survey allowed for examination of sleep, mental health, and child, family, and neighborhood factors together.

This study also has several limitations. The cross-sectional analysis did not allow examination of causal pathways or bidirectional influences of sleep duration and risk factors. Parent-reported data may be affected by recall bias, social desirability, or parents’ interpretation of items. The question for the sleep item had a minor wording change for children aged 6 to 17 years beginning with the 2018 survey. Although sensitivity analyses showed similar estimates and the same patterns of effects before and after the question was changed, this change should be considered when interpreting the results. The sleep indicator in this study measured a single aspect of healthy sleep, duration, and did not assess quality of sleep, presence of sleep disorders, and sleep architecture, all of which affect the restorative function of sleep (5,8). As mentioned, parents may overestimate sleep duration — possibly because parent reports often describe time in bed rather than time actually asleep — particularly for older children and adolescents, leading to lower-than-expected estimates for short sleep (32). Without objective measures, such as actigraphy or polysomnography, the association between insufficient sleep and mental health while accounting for community and family factors cannot be fully described (22).

The contextual factors included in this study do not cover the breadth of possible influences on sleep. Other related factors may affect sleep, such as the quality of the parent–child relationship or perinatal risks, among others (12,15). Moreover, school and community factors, such as later school start times and time spent in extracurricular activities, affect sleep duration and are influenced by cultural context, which in turn can influence family factors such as enforcing consistent bedtimes (20).

The data in our study were collected before the COVID-19 pandemic, which has had positive and negative effects on factors related to adolescent sleep and mental health, such as more outdoor time and later school start times but also more stress, disruption of routines, and increased screen time (28,33). The long-term effects of the pandemic on sleep and mental health cannot yet be determined.

### Implications for intervention

This study provides information to support that short sleep duration is a widespread public health concern among children of all ages. Many risk factors contribute independently to short sleep duration and present opportunities for intervention, for example, improving bedtime routines. Sleep education and behavioral health interventions may be feasible and effective (3). Parents and older children and adolescents may have misconceptions about sleep recommendations, and these misconceptions may affect sleep hygiene and sleep duration (19,24). This population may benefit from learning behaviors that promote healthy sleep, such as avoiding electronics and caffeine before bedtime (3,5). Health care providers can receive training to educate families about healthy sleep routines (1,3,5,9). Expectations about sleep and the effect of sleep interventions may be influenced by child age, family context, and cultural factors; more information could inform developmentally and culturally sensitive approaches (5,24,26,32). However, consistent bedtimes and sufficient time in bed are only 2 components of healthy sleep (8,19); health care providers can provide treatment to address other sleep problems that reduce healthy sleep, such as insomnia or obstructive sleep apnea (5,13). Addressing sleep concerns may be especially important for children with mental and developmental disorders (5,18).

Healthy sleep can also be promoted in schools and early care and education through educating children and families, and through policies and procedures that encourage sufficient sleep (3). Public health attention to the decline of sleep duration among adolescents in past decades has resulted in strategies such as later high school start times (3). Given that short sleep duration affected children of all ages and was highest in children aged 6 to 12 years, schools can also evaluate school start times for elementary and middle school and address other school policies, such as amount of homework and time required for extracurricular activities, that could affect children’s ability to get sufficient sleep (20).

Our findings show risks and disparities related to child sleep on the individual, family, and community level. Attention to sleep as a risk for negative outcomes may be particularly important among children with MBDD. Conversely, addressing children’s mental health concerns and improving factors that may influence mental health may result in better sleep. For example, in families where stress may interfere with sleep, parents, particularly those with poor mental or physical health, may benefit from receiving additional support for themselves and to strengthen the parent–child relationship, which may act as a buffer against poor mental health in racial and ethnic minority groups (12). Parents of children with sleep concerns, particularly if co-occurring with other disorders, may also need support to achieve sufficient sleep themselves (18).

Sleep interventions can be tailored to people who are most affected and address risks and disparities on each level — from individual to systemic (9,10). The historical decline in sleep duration disproportionately affects communities that experience other health disparities such as racial and ethnic minority groups, students living in urban areas, and those of low socioeconomic status (24), thus contributing to cumulative risk. Intervention approaches may focus on communities at risk by introducing sleep hygiene education in affordable housing communities, offering sleep education, and improving sleep hygiene by supplying beds for children and adolescents (9).

On a community level, interventions can also address improving the neighborhood context (17). Increasing safe access to green space and recreational areas such as parks and community gardens may directly and indirectly improve sleep by encouraging physical activity; reducing lights, noise, and temperature; and decreasing stress by facilitating social cohesion (9,30). Implementing public health policies to reduce crime and increase community safety can address structural inequalities and potentially ameliorate sleep disparities (9,30). Combining public health interventions that focus on improving sleep among children by addressing risks at the child, family, and community level may lead to improved long-term health, development, and well-being (2).

## References

[1] Paruthi S , Brooks LJ , D’Ambrosio C , Hall WA , Kotagal S , Lloyd RM , Consensus statement of the American Academy of Sleep Medicine on the recommended amount of sleep for healthy children: methodology and discussion. J Clin Sleep Med 2016;12(11):1549–61. 10.5664/jcsm.6288 27707447 PMC5078711

[2] Itani O , Jike M , Watanabe N , Kaneita Y . Short sleep duration and health outcomes: a systematic review, meta-analysis, and meta-regression. Sleep Med 2017;32:246–56. 10.1016/j.sleep.2016.08.006 27743803

[3] Ramar K , Malhotra RK , Carden KA , Martin JL , Abbasi-Feinberg F , Aurora RN , Sleep is essential to health: an American Academy of Sleep Medicine position statement. J Clin Sleep Med 2021;17(10):2115–9. 10.5664/jcsm.9476 34170250 PMC8494094

[4] Astill RG , Van der Heijden KB , Van Ijzendoorn MH , Van Someren EJW . Sleep, cognition, and behavioral problems in school-age children: a century of research meta-analyzed. Psychol Bull 2012;138(6):1109–38. 10.1037/a0028204 22545685

[5] Meltzer LJ , Williamson AA , Mindell JA . Pediatric sleep health: It matters, and so does how we define it. Sleep Med Rev 2021;57:101425. 10.1016/j.smrv.2021.101425 33601324 PMC9067252

[6] Alfano CA , Bower JL , Harvey AG , Beidel DC , Sharp C , Palmer CA . Sleep restriction alters children’s positive emotional responses, but effects are moderated by anxiety. J Child Psychol Psychiatry 2020;61(10):1150–9. 10.1111/jcpp.13287 32621796

[7] Wheaton AG , Claussen AH . Short sleep duration among infants, children, and adolescents aged 4 months–17 years — United States, 2016–2018. MMWR Morb Mortal Wkly Rep 2021;70(38):1315–21. 10.15585/mmwr.mm7038a1 34555000 PMC8459893

[8] Dutil C , Walsh JJ , Featherstone RB , Gunnell KE , Tremblay MS , Gruber R , Influence of sleep on developing brain functions and structures in children and adolescents: a systematic review. Sleep Med Rev 2018;42:184–201. 10.1016/j.smrv.2018.08.003 30241996

[9] Billings ME , Cohen RT , Baldwin CM , Johnson DA , Palen BN , Parthasarathy S , Disparities in sleep health and potential intervention models: a focused review. Chest 2021;159(3):1232–40. 10.1016/j.chest.2020.09.249 33007324 PMC7525655

[10] Johnson DA , Billings ME , Hale L . Environmental determinants of insufficient sleep and sleep disorders: implications for population health. Curr Epidemiol Rep 2018;5(2):61–9. 10.1007/s40471-018-0139-y 29984131 PMC6033330

[11] Heissel JA , Sharkey PT , Torrats-Espinosa G , Grant K , Adam EK . Violence and vigilance: the acute effects of community violent crime on sleep and cortisol. Child Dev 2018;89(4):e323–31. 10.1111/cdev.12889 28741650 PMC5783802

[12] So M , Perry NB , Langenfeld AD , Barnes AJ . Adolescent sleep and mental health across race/ethnicity: does parent–child connectedness matter? J Dev Behav Pediatr 2021;42(9):742–50. 10.1097/DBP.0000000000000958 34840318

[13] Acosta J , Parent J , DiMarzio K , McMakin DL , McKee LG , Dale CF . Longitudinal associations between parenting practices and youth sleep problems. J Dev Behav Pediatr 2021;42(9):751–60. 10.1097/DBP.0000000000000953 33908375 PMC8548445

[14] Bøe T , Hysing M , Stormark KM , Lundervold AJ , Sivertsen B . Sleep problems as a mediator of the association between parental education levels, perceived family economy and poor mental health in children. J Psychosom Res 2012;73(6):430–6. 10.1016/j.jpsychores.2012.09.008 23148810

[15] Dai Y , Trout KK , Liu J . Perinatal physiological and psychological risk factors and childhood sleep outcomes: a systematic review and meta-analysis. J Dev Behav Pediatr 2022;43(9):e629–44. 10.1097/DBP.0000000000001123 36067425 PMC10002289

[16] DeSantis A , Troxel WM , Beckman R , Ghosh-Dastidar B , Hunter GP , Hale L , Is the association between neighborhood characteristics and sleep quality mediated by psychological distress? An analysis of perceived and objective measures of 2 Pittsburgh neighborhoods. Sleep Health 2016;2(4):277–82. 10.1016/j.sleh.2016.08.001 28393098 PMC5380919

[17] Dubowitz T , Haas A , Ghosh-Dastidar B , Collins RL , Beckman R , Brooks Holliday S , Does investing in low-income urban neighborhoods improve sleep? Sleep 2021;44(6):zsaa292. 10.1093/sleep/zsaa292 33417708 PMC8193558

[18] Varma P , Conduit R , Junge M , Lee VV , Jackson ML . A systematic review of sleep associations in parents and children. J Child Fam Stud 2021;30(9):2276–88. 10.1007/s10826-021-02002-5

[19] Jarrin DC , Abu Awad Y , Rowe H , Noel NAO , Ramil J , McGrath JJ . Parental expectations are associated with children’s sleep duration and sleep hygiene habits. J Dev Behav Pediatr 2020;41(7):550–8. 10.1097/DBP.0000000000000818 32433218

[20] Short MA , Gradisar M , Lack LC , Wright HR , Dewald JF , Wolfson AR , A cross-cultural comparison of sleep duration between US and Australian adolescents: the effect of school start time, parent-set bedtimes, and extracurricular load. Health Educ Behav 2013;40(3):323–30. 10.1177/1090198112451266 22984209 PMC4232364

[21] Grandner MA , Williams NJ , Knutson KL , Roberts D , Jean-Louis G . Sleep disparity, race/ethnicity, and socioeconomic position. Sleep Med 2016;18:7–18. 10.1016/j.sleep.2015.01.020 26431755 PMC4631795

[22] Tomfohr-Madsen L , Cameron EE , Dhillon A , MacKinnon A , Hernandez L , Madigan S , Neighborhood socioeconomic status and child sleep duration: a systematic review and meta-analysis. Sleep Health 2020;6(5):550–62. 10.1016/j.sleh.2020.02.012 32335039

[23] El-Sheikh M , Gillis BT , Saini EK , Erath SA , Buckhalt JA . Sleep and disparities in child and adolescent development. Child Dev Perspect 2022;16(4):200–7. 10.1111/cdep.12465 36337834 PMC9629655

[24] Keyes KM , Maslowsky J , Hamilton A , Schulenberg J . The great sleep recession: changes in sleep duration among US adolescents, 1991–2012. Pediatrics 2015;135(3):460–8. 10.1542/peds.2014-2707 25687142 PMC4338325

[25] US Census Bureau. National Survey of Children’s Health (NSCH). Page last revised March 31, 2023. Accessed April 5, 2023. https://www.census.gov/programs-surveys/nsch.html

[26] US Census Bureau. Methodology and data user FAQs. Page last revised November 19, 2021. Accessed November 25, 2023. https://www.census.gov/programs-surveys/nsch/technical-documentation/methodology.html

[27] Parker JD , Talih M , Malec DJ , Beresovsky V , Carroll M , Gonzalez JF , National Center for Health Statistics data presentation standards for proportions. Vital Health Stat 2 2017;(175):1–22. 30248016

[28] Wheaton AG , Jones SE , Cooper AC , Croft JB . Short sleep duration among middle school and high school students — United States, 2015. MMWR Morb Mortal Wkly Rep 2018;67(3):85–90. 10.15585/mmwr.mm6703a1 29370154 PMC5812312

[29] Guglielmo D , Gazmararian JA , Chung J , Rogers AE , Hale L . Racial/ethnic sleep disparities in US school-aged children and adolescents: a review of the literature. Sleep Health 2018;4(1):68–80. 10.1016/j.sleh.2017.09.005 29332684 PMC5771439

[30] Singh GK , Kenney MK . Rising prevalence and neighborhood, social, and behavioral determinants of sleep problems in US children and adolescents, 2003–2012. Sleep Disord 2013;2013:394320. 10.1155/2013/394320 23819057 PMC3683488

[31] Tyack C , Unadkat S , Voisnyte J . Adolescent sleep — lessons from COVID-19. Clin Child Psychol Psychiatry 2022;27(1):6–17. 10.1177/13591045211065937 34935514 PMC8829149

[32] Hirshkowitz M , Whiton K , Albert SM , Alessi C , Bruni O , DonCarlos L , National Sleep Foundation’s sleep time duration recommendations: methodology and results summary. Sleep Health 2015;1(1):40–3. 10.1016/j.sleh.2014.12.010 29073412

[33] Kiss O , Alzueta E , Yuksel D , Pohl KM , de Zambotti M , Műller-Oehring EM , The pandemic’s toll on young adolescents: prevention and intervention targets to preserve their mental health. J Adolesc Health 2022;70(3):387–95. 10.1016/j.jadohealth.2021.11.023 35090817 PMC8789404

